# Association of sputum microbiota profiles with severity of community-acquired pneumonia in children

**DOI:** 10.1186/s12879-016-1670-4

**Published:** 2016-07-08

**Authors:** Melinda M. Pettigrew, Janneane F. Gent, Yong Kong, Martina Wade, Shane Gansebom, Anna M. Bramley, Seema Jain, Sandra L. R. Arnold, Jonathan A. McCullers

**Affiliations:** Yale School of Public Health, New Haven, CT USA; Yale School of Medicine, New Haven, CT USA; St. Jude Children’s Research Hospital, Memphis, TN USA; Centers for Disease Control and Prevention, Atlanta, GA USA; University of Tennessee Health Science Center, Memphis, TN USA; Department of Epidemiology of Microbial Diseases, Yale School of Public Health, 60 College Street, LEPH 720, New Haven, CT 06515 USA

**Keywords:** Microbiota, Microbial communities, Respiratory tract, Pneumonia, Children

## Abstract

**Background:**

Competitive interactions among bacteria in the respiratory tract microbiota influence which species can colonize and potentially contribute to pathogenesis of community-acquired pneumonia (CAP). However, understanding of the role of respiratory tract microbiota in the clinical course of pediatric CAP is limited.

**Methods:**

We sought to compare microbiota profiles in induced sputum and nasopharyngeal/oropharyngeal (NP/OP) samples from children and to identify microbiota profiles associated with CAP severity. We used 16S ribosomal RNA sequencing and several measures of microbiota profiles, including principal component analysis (PCA), to describe the respiratory microbiota in 383 children, 6 months to <18 years, hospitalized with CAP. We examined associations between induced sputum and NP/OP microbiota profiles and CAP severity (hospital length of stay and intensive care unit admission) using logistic regression.

**Results:**

Relative abundance of bacterial taxa differed in induced sputum and NP/OP samples. In children 6 months to < 5 years, the sputum PCA factor with high relative abundance of *Actinomyces*, *Veillonella*, *Rothia,* and Lactobacillales was associated with decreased odds of length of stay ≥ 4 days [adjusted odds ratio (aOR) 0.69; 95 % confidence interval (CI) 0.48–0.99]. The sputum factor with high relative abundance of *Haemophilus* and Pasteurellaceae was associated with increased odds of intensive care unit admission [aOR 1.52; 95 % CI 1.02–2.26]. In children 5 to < 18 years, the sputum factor with high relative abundance of Porphyromonadaceae, Bacteriodales, Lactobacillales, and *Prevotella* was associated with increased odds of length of stay ≥ 4 days [aOR 1.52; 95 % CI 1.02–2.26]. Taxa in NP/OP samples were not associated with CAP severity.

**Conclusion:**

Certain taxa in the respiratory microbiota, which were detected in induced sputum samples, are associated with the clinical course of CAP.

**Electronic supplementary material:**

The online version of this article (doi:10.1186/s12879-016-1670-4) contains supplementary material, which is available to authorized users.

## Background

Community-acquired pneumonia (CAP) is an important cause of morbidity and mortality in children [1, 2]. Traditional models of CAP pathogenesis have described this disease as resulting from invasion and growth of a single pathogen in the lung (e.g., *Streptococcus pneumoniae* as the etiologic agent); and epidemiologic studies frequently seek to identify a limited number of bacteria and respiratory viruses as the causative agents of CAP [3, 4]. However, newer models of CAP pathogenesis posit that the upper and lower airways are a complex and connected ecosystem and that CAP occurs as a consequence of disruptions in respiratory tract homeostasis [5]. Few, if any, culture-independent studies have examined associations between the clinical course of CAP in hospitalized children and samples from both the upper and lower respiratory tract.

CAP results from complex interactions between the host immune response, the respiratory tract microbiota, and CAP-associated pathogens [5–7]. Competitive interactions among bacteria of the upper respiratory tract microbiota influence which species can colonize the respiratory tract and potentially contribute to pathogenesis of CAP [8–11]. Bacteria within the upper and lower respiratory tract microbiota can alter host immune responses and the virulence of CAP pathogens [12–14]. Respiratory virus infection may lead to the overgrowth of select bacteria in the upper respiratory tract microbiota, facilitate transition of bacteria into the lower airways, or promote enhanced replication of bacteria already present in the lower airways [1, 15–18]. Collectively, these data suggest that the composition of the respiratory tract microbiota influences the clinical course of CAP.

The Centers for Disease Control (CDC) Etiology of Pneumonia in the Community (EPIC) study was a prospective, multi-site, population-based active surveillance study to determine the incidence and etiology of CAP in the United States [4]. Nasopharyngeal (NP), oropharyngeal (OP), and induced sputum samples were collected from children at the Memphis, TN site. The value of respiratory tract samples for detection of bacterial CAP pathogens in children has been debated [19, 20]. Culture data from induced sputum samples have been shown to provide useful information in children with pneumonia [20] and cystic fibrosis [21]. While sputum samples have limitations, we hypothesized that they would provide relevant information regarding microbial environment of the lower airways in children with CAP [20, 22, 23]. Our overall goal was to describe the respiratory tract microbiota in children hospitalized with CAP. Specifically we sought to 1) use 16S ribosomal RNA (rRNA) microbial profiling to determine whether microbiota profiles differ between induced sputum and NP/OP samples from children hospitalized with CAP, and 2) identify microbiota profiles associated with CAP severity as measured by length of hospital stay (LOS) and intensive care unit (ICU) admission.

## Methods

### Study design and participants

Data for the current study were collected at Le Bonheur Children’s Hospital, the Memphis, TN site of the EPIC study [4]. The institutional review boards at the University of Tennessee Health Science Center and the CDC approved the study. Written informed consent was obtained from parents or caregivers prior to enrollment and children provided assent when age appropriate. This analysis focused on a convenience sample of children hospitalized with radiographically confirmed CAP, including only those who were enrolled in the first two years of the EPIC study, between January 2010 and December 2011. A detailed description of the EPIC study methods is provided elsewhere [4]. We restricted our study population to 383 children who were ≥ 6 months to < 18 years of age and contributed an induced sputum, NP, and OP sample. Children < 6 months were excluded due to differences in the associated pathogens and severity of CAP in neonates and very young infants [24].

### Sample collection, clinical and epidemiologic data

Induced sputum, NP, and OP samples were collected at enrollment: 89 % within 24 h of hospital admission and 11 % between 24 and 48 h of hospital admission. Sputum production was induced via inhalation of albuterol followed by 7 % saline to induce deep cough [20]. Suctioning through the nose or mouth was then used in children who were too young to expectorate. Trained staff obtained an NP and an OP swab from each of the enrolled children.

Sputum samples were plated for culture of CAP associated bacterial pathogens (e.g., *S. pneumoniae*, *Haemophilus influenzae*, *Moraxella cattharalis*, and *Staphylcococcus aureus*) using standard microbiologic methods. Two hundred microliters of the original sputum sample was diluted in 1 mL of EasyMag lysis buffer and stored at -80 °C until DNA extraction. DNA extraction of sputum samples was performed using the Biomerieux NucliSENS EasyMag instrument. NP and OP swabs were combined to maximize detection of respiratory viruses and are hereafter referred to as NP/OP swabs. DNA was extracted from NP/OP swabs using the Maxwell® LEV Blood DNA kit (Madison, WI) with minor modifications; samples were centrifuged at 7500 rpm for 10 min and the pellet resuspended in 180 μl of lysis solution containing mutanolysin, lysozyme, and lysostaphin. The sample was incubated for 30 min at 37 °C and then processed according to the manufacturer’s instructions.

Etiologic data from the EPIC study included results from diagnostic testing for bacterial pathogens by culture of blood or other specimens (e.g., pleural fluid) and PCR of whole blood to identify *S. pneumoniae* and group A streptococcus [4]. NP/OP swabs were tested by PCR using CDC primers for *Mycoplasma pneumoniae*, *Chlamydophila pneumoniae,* and the following respiratory viruses: human rhinovirus, respiratory syncytial virus (RSV), adenovirus, human metapneumovirus, coronavirus (types OC43, 229E, NL63, and HKU1), parainfluenza viruses (types 1–3), and influenza A and B [4]. With the exception of rhinovirus and coronaviruses, serologic tests were performed for the previously listed viruses.

Data regarding demographic characteristics, underlying conditions, and exposures known to alter the composition of the respiratory microbiota (e.g., prior antibiotic use, fall/winter season) [10, 25–27] were also obtained in the EPIC study [4]. Demographic data, environmental tobacco smoke exposure, and prior antibiotic use data were collected via child/caregiver report. Environmental tobacco smoke exposure was categorized as present if smoking was allowed in the child’s home. Prior antibiotic use was defined as any use of antibiotics prior to hospitalization for the current CAP episode or chronic use of antibiotics. Presence of asthma/reactive airway disease and chronic conditions other than asthma were determined by child/caregiver report. Chronic conditions other than asthma included congenital heart disease, chronic lung disease, diabetes, kidney, heart or liver disorder, immunosuppression, and pre-term birth. Additional data obtained through medical chart abstraction included LOS (defined as the length of time in days from the admission date, or triage date if applicable, to hospital discharge) and ICU admission. Data on vaccination for *H. influenzae* type B and *S. pneumoniae* were collected via child/caregiver report and verified primarily by obtaining vaccine records from primary care physicians and by reviewing vaccine registries. Vaccines have been shown to alter the nasopharyngeal flora [26]. Thus, children were classified as vaccinated for *H. influenzae* type B (yes/no) or *S. pneumoniae* (yes/no) if at least one vaccine dose was received prior to enrollment and verified. This study population included older children who may not have received the pneumococcal vaccine because they were either born before, or were older than, the recommended age for vaccination when pneumococcal conjugate vaccines became available.

### Sequencing and processing of 16S rRNA gene sequence reads

PCR amplification of the 16S rRNA hypervariable region V4 was performed on extracted DNA samples in duplicate with negative controls using 515 F and 806R barcoded primers [28]. Samples were pooled in equimolar amounts in batches of up to 96 samples. Pooled PCR products were sent to the Yale Center for Genome Analysis using the Illumina MiSeq platform [28].

*Btrim* software was used to sort, trim, and filter low quality sequences [10, 29]. Cleaned and filtered sequences were processed using customized analytic pipelines and open source packages available through the Ribosomal Database Project (RDP) [10, 11, 30]. Sequence reads were aligned using the RDP alignment tool. The command line RDP classifier tool was used for taxonomic assignment [30]. Sequences were classified in an iterative process. Taxa were defined by grouping together all sequences belonging to the same genus. Sequences that were unclassified at the genus level were classified and then grouped at the next lowest taxonomic level.

### Characterization of microbiota profiles

We used three different approaches to describe microbiota profiles. First, we examined the distribution of the relative abundance of each taxon more frequent than 0.8 % of the microbial community (*n* = 22 taxa). The distributions for relative abundances were highly skewed; therefore, we identified the top quartile of the distribution within each age group. Cutoff values for the 75^th^ percentile are shown in Additional file 1: Table S1. We then created a binary variable for each sample taxon as being in the top quartile of the distribution or not. Second, we identified individual taxa that were differentially abundant in groups of samples using linear discriminant analysis (LDA) effect size (LefSe) [31]. We used 2.0 as the threshold cutoff value on the logarithmic LDA score for identifying taxa that differed in abundance between comparison groups [31]. Taxa within the airway microbiota may co-colonize based on similar nutritional requirements or utilization of secondary metabolites [32]. Thus, for our third approach, we used principal component analysis (PCA) to examine relationships among the same 22 taxa identified above. PCA is a statistical method used to identify groups of correlated variables within a dataset (e.g., bacterial taxa) [33]. These subsets of correlated variables are then combined into independent factors that represent linear relationships among taxa. The factors are interpreted as reflecting underlying processes that created the observed associations. We specified an eigenvalue of one and an orthogonal rotation in the PCA. Taxa with a loading value of at least ± 0.4 were retained and considered taxa of interest for that factor [10, 11, 33].

### Statistical analysis

All analyses were stratified by age categorized as 6 months to < 5 years and 5 to < 18 years. CAP severity was measured by 1) LOS ≥ 4 days (yes/no), the 75^th^ percentile of the distribution of LOS, and 2) ICU admission (yes/no). To explore associations between measures of microbiota profiles and CAP severity, we used multivariate logistic regression. In order to identify potential confounders, we evaluated unadjusted associations between individual patient characteristics and exposures of interest (e.g., taxa identified by LefSe as differentially abundant between groups) and outcomes (i.e., LOS or ICU admission) by chi-square test, Fisher’s exact test, and analysis of variance or paired *t*-tests as appropriate. Taxa identified by LefSe as differentially present in the CAP severity groups (LOS < 4 or ≥ 4 days or ICU admission (yes/no)) were categorized as being in the top quartile of that taxon’s distribution and included in the models as a binary variable (yes/no). Factor scores from PCA were included in models as continuous variables. Models were adjusted for potential confounders if they were associated with the exposure and the outcome (*P* < 0.15) in unadjusted analyses [34]. Variables were removed if they were not statistically significant (*P* < 0.05) and if removal did not change the likelihood ratio by more than 10 %. Statistical analyses were done using SAS 9.3 and R version 3.0.1 [35].

## Results

### Patient characteristics

Demographic and clinical characteristics of the children hospitalized with CAP are shown in Table 1. Of the 383 children, 68.7 % were 6 months to < 5 years of age and 79.1 % were black. Over half (53.0 %) had a history of asthma/reactive airway disease and nearly a quarter (24.0 %) had chronic conditions other than asthma (Table 1). A history of asthma/reactive airway disease and ICU admission were more frequent in children 5 to < 18 years compared to children 6 months to < 5 years of age. The majority of children received at least one dose of pneumococcal conjugate vaccine (91.6 %). Fewer children 5 to < 18 years received at least one dose of pneumococcal conjugate vaccine compared to children 6 months to <5 years of age.

**Table 1:** Characteristics of 383 children, 6 months to <18 years of age, hospitalized with community-acquired pneumonia

		Age group		
		6 months to <5 years	5 to <18 years	
Characteristic	*N* (%)	%	%	*P* value
Age				
6 months to < 5 years	263 (68.7)	--	--	
5 to <18 years	120 (31.3)	--	--	
Race/Ethnicity				0.25
White	47 (12.3)	10.6	15.8	
Black	303 (79.1)	79.8	77.5	
Other	33 (8.6)	6.1	2.5	
Gender				0.43
Female	171 (44.6)	46.0	41.7	
Male	212 (55.4)	54.0	58.3	
Fall/winter season	225 (58.8)	59.7	56.7	0.58
Environmental tobacco smoke exposure^a^	44 (11.6)	10.4	14.2	0.29
Medical history				
Asthma/reactive airway disease	203 (53.0)	47.9	64.2	**0.003**
Chronic conditions other than asthma	92 (24.0)	24.0	24.2	0.96
Prior Antibiotics	82 (21.4)	22.4	19.2	0.47
Pneumococcal vaccine^b^	347 (91.6)	98.5	76.5	**<0.0001**
Length of stay (days)				0.18
4 or more	105 (27.4)	24.3	34.2	
ICU admission	50 (13.0)	9.9	20.0	**0.006**

Numbers in the columns in each group represent the percent of children with that characteristic in that age group. Bold indicates a significant *P* value of <0.05

^a^Missing data for 4 subjects

^b^Receipt of at least one or more doses prior to enrollment, missing data for 4 subjects

Diagnostic testing resulted in detection of *S. pneumoniae* in nine (2.4 %) children and *H. influenzae* in one (0.3 %) (Additional file 1: Table S2). One or more respiratory viruses were identified in 310 (80.9 %) children (Additional file 1: Table S2). Rhinovirus and respiratory syncytial virus (RSV) were the two most common respiratory viruses detected (Additional file 1: Table S2). Subsequent analyses of bacteria and viruses detected through diagnostic testing were restricted to rhinovirus and RSV due to the low frequency of detection of other CAP pathogens. RSV was detected more frequently in children 6 months to <5 years whereas rhinovirus was detected more frequently in children 5 to <18 years (Additional file 1: Table S2). Sputum culture results were not used for etiologic diagnosis and are included in Additional file 1: Table S2. *S. pneumoniae*, *H. influenzae* and *M. catarrhalis* were detected more frequently in children 6 months to <5 years than in children 5 to <18 years. Subsequent analyses were stratified by age group due to the age difference in prevalence of bacteria and respiratory viruses in this study population (Additional file 1: Table S2), and previously described differences in the prevalence of colonization by potential CAP pathogens and CAP severity by age [24, 36, 37].

### Comparison of sputum and NP/OP samples

A mean (standard deviation [SD]) of 86,879 (37,698) sequence reads were obtained in sputum samples. A mean of 92,534 (32,060) sequence reads were obtained in NP/OP samples.

*Streptococcus*, *Moraxella,* or *Haemophilus* was the dominant taxon in individual sputum samples for the majority of children 6 months to <5 years and in individual NP/OP samples for the majority of children of any age (Table 2 and Additional file 2: Figure S1). In contrast, *Streptococcus*, *Prevotella*, or Pasteurellaceae was the dominant taxon in individual sputum samples for the majority of children 5 to <18 years (Table 2 and Additional file 2: Figure S1).

**Table 2:** Proportion of children with a given dominant bacterial taxon by age group, 6 months to <5 years (*n* = 263) or 5 to <18 years (*n* = 120)

	6 months to <5 years		5 to <18 years	
	Sputum	NP/OP	Sputum	NP/OP
Taxa	N (%)	N (%)	N (%)	N (%)
*Streptococcus* sp.	112 (42.6)	188 (71.5)	50 (41.7)	58 (48.3)
*Moraxella* sp.	60 (22.8)	19 (7.2)	3 (2.5)	30 (25.0)
*Haemophilus* sp.	36 (13.7)	22 (8.4)	4 (3.3)	10 (8.3)
*Prevotella* sp.	20 (7.6)	3 (1.1)	38 (31.7)	2 (1.7)
*Pasteurellaceae* family	16 (6.1)	9 (3.4)	6 (5)	2 (1.7)
*Neisseria* sp.	7 (2.7)	10 (3.8)	4 (3.3)	(0)
*Fusobacterium* sp.	3 (1.1)	(0)	2 (1.7)	1 (0.8)
*Veillonella* sp.	3 (1.1)	5 (1.9)	2 (1.7)	1 (0.8)
*Corynebacterium* sp.	1 (0.4)	(0)	(0)	2 (1.7)
*Dolosigranulum* sp.	1 (0.4)	(0)	1 (0.8)	(0)
*Leptotrichia* sp.	1 (0.4)	(0)	1 (0.8)	2 (1.7)
*Mycoplasma* sp.	1 (0.4)	(0)	6 (5)	(0)
*Rothia* sp.	1 (0.4)	2 (0.8)	1 (0.8)	3 (2.5)
*Porphyromonadaceae* family	1 (0.4)	1 (0.4)	1 (0.8)	5 (4.2)
*Actinomyces* sp.	(0)	(0)	1 (0.8)	(0)
*Gemella* sp.	(0)	2 (0.8)	(0)	1 (0.8)
*Bacteroidetes* phylum	(0)	2 (0.8)	(0)	3 (2.5)

**Fig. 1:**
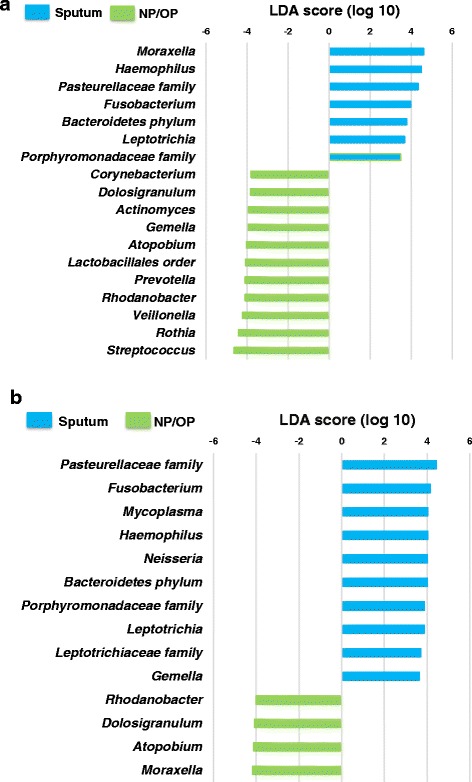
Differences in the abundance of taxa in sputum and nasopharyngeal/oropharyngeal (NP/OP) samples from children 6 months to <5 years (**a**) and 5 to <18 years (**b**). Differences were identified using linear discriminant analysis (LDA) effect size (LefSe). A threshold of 2.0 on the logarithmic LDA score was used to identify taxa that significantly differed in abundance between sputum and NP/OP samples

LefSe indicated that taxa within sputum and NP/OP samples differ in their relative abundance. Among children 6 months to <5 years, the relative abundance of seven taxa was higher in sputum samples and the relative abundance of 11 taxa was higher in NP/OP samples (Fig. 1a). Among children 5 to <18 years, the relative abundance of 10 taxa was higher in sputum samples and the relative abundance of four taxa was higher in NP/OP samples (Fig. 1b).

### Unadjusted associations between individual characteristics and CAP outcomes

Unadjusted associations between individual characteristics and LOS ≥4 days or ICU admission are shown in Table 3. In children 6 months to <5 years, the distributions of patient characteristics and season of enrollment were similar among those with LOS <4 days and those with LOS ≥4 days and among those with and without ICU admission (Table 3). However, RSV was more frequently detected in children who did not (43.9 %) vs. children who did (23.1 %) have an ICU admission (*P* = 0.04) (Table 3). Among children 5 to <18 years, asthma/reactive airway disease was more frequent in children with LOS <4 days compared to those with LOS ≥4 days (73.4 % vs. 46.3 % respectively, *P* = 0.003) (Table 3). Receipt of at least one dose of pneumococcal conjugate vaccine was more frequent in those with LOS <4 days compared to those with LOS ≥4 days (83.5 % vs. 62.5 % respectively, *P* = 0.01) (Table 3).

**Table 3:** Unadjusted associations between individual characteristics [n (%)] of children 6 months to <5 years (*n* = 263) or 5 to <18 years (*n* = 120), hospitalized with community-acquired pneumonia and length of stay (LOS) or intensive care unit (ICU) admission

		LOS			ICU admission		
		<4 days	≥ 4 days		No	Yes	
Characteristic	N (%)	(%)	(%)	*P* value	(%)	(%)	*P* value
Age 6 months to <5 years							
Demographic							
Race/Ethnicity				0.35			0.64
White	28 (10.6)	10.6	10.9		10.1	15.4	
Black	210 (79.8)	81.4	75.0		80.6	73.1	
Other	25 (9.5)	8.0	14.1		9.3	11.5	
Gender				0.87			0.67
Female	121 (46.0)	45.7	46.9		45.6	50.0	
Male	124 (54.0)	54.3	53.1		54.4	50.0	
Fall/winter season	157 (59.7)	59.3	60.9	0.82	60.7	50.0	0.29
Environmental tobacco smoke exposure	27 (10.4)	11.8	6.3	0.21	9.9	15.4	0.32
Medical History							
Asthma/reactive airway disease	126 (47.9)	44.7	57.8	0.07	46.8	57.7	0.29
Chronic conditions other than asthma	63 (24.0)	22.1	29.7	0.22	23.6	26.9	0.71
Prior antibiotics	59 (22.4)	22.6	21.9	0.90	22.4	23.1	0.93
Pneumococcal vaccine^a^	256 (98.5)	98.5	98.4	0.97	98.7	96.0	0.29
Respiratory virus positive							
RSV	110 (41.8)	42.7	39.1	0.61	43.9	23.1	**0.04**
Rhinovirus	89 (33.8)	36.2	26.6	0.16	35.4	19.2	0.10
Age 5 to <18 years							
Demographic							
Race/Ethnicity				0.81			0.73
White	19 (15.8)	16.5	14.6		16.7	12.5	
Black	93 (77.5)	76.0	80.5		76.0	83.3	
Other	8 (6.7)	7.6	4.9		7.3	4.2	
Gender				0.05			0.16
Female	50 (41.7)	35.4	53.7		38.5	54.2	
Male	70 (58.3)	64.6	46.3		61.5	45.8	
Fall/winter season	68 (56.7)	58.2	53.7		57.3	54.2	0.78
Environmental tobacco smoke exposure	17 (14.2)	15.2	12.2	0.66	14.6	12.5	1.00
Medical History							
Asthma/reactive airway disease	77 (64.2)	73.4	46.3	**0.003**	64.6	62.5	0.85
Chronic conditions other than asthma	29 (24.2)	19.0	34.2	0.07	20.8	37.5	0.09
Prior antibiotics	23 (19.2)	16.5	24.4	0.30	19.8	16.7	0.73
Pneumococcal vaccine^b^	91 (76.5)	83.5	62.5	**0.01**	80.2	60.9	0.05
Respiratory virus positive							
RSV	13 (10.8)	12.7	7.3	0.37	9.4	16.7	0.30
Rhinovirus	59 (49.2)	55.7	36.6	0.05	46.9	58.3	0.32

^a^Receipt of at least one or more doses prior to enrollment, missing data for 3 subjects. Bold indicates a significant *P* value of <0.05

^b^Receipt of at least one or more doses prior to enrollment, missing data for 1 subject

### Adjusted associations between sputum microbiota profiles and CAP outcomes

Results for all final, adjusted logistic regression models are given below for each of our methods of describing microbiota profiles: individual taxa and PCA factors. In children 6 months to <5 years, final models of the association between microbiota profiles and LOS ≥4 days were adjusted for asthma/reactive airway disease and models of the association between microbiota profiles and ICU admission were adjusted for the presence of rhinovirus and RSV. In children 5 to <18 years, final models of the association between microbiota profiles and LOS ≥4 days vs. < 4 days were adjusted for asthma/reactive airway disease, chronic conditions other than asthma, and gender, and models of the association between microbiota profiles and ICU admission were adjusted for chronic conditions other than asthma.

#### Individual taxa in sputum

We identified individual taxa that were differentially abundant in CAP severity groups using LefSe. We then evaluated each taxon separately in adjusted models. In children 6 months to <5 years, high relative abundance of *Actinomyces* [aOR 0.29; 95 % confidence interval (CI) 0.12–0.67], *Veillonella* [aOR 0.40, 95 % CI 0.18–0.86], and *Rothia* [aOR 0.33, 95 % CI 0.15–0.75] were individually associated with decreased odds of LOS ≥4 days. In children 5 to <18 years, high relative abundance of *Gemella* was associated with decreased odds of LOS ≥4 days [aOR 0.05; 95 % CI 0.01–0.39]. There were no taxa that were individually associated with ICU admission in adjusted models in either age group.

#### PCA factors in sputum

**Fig. 2:**
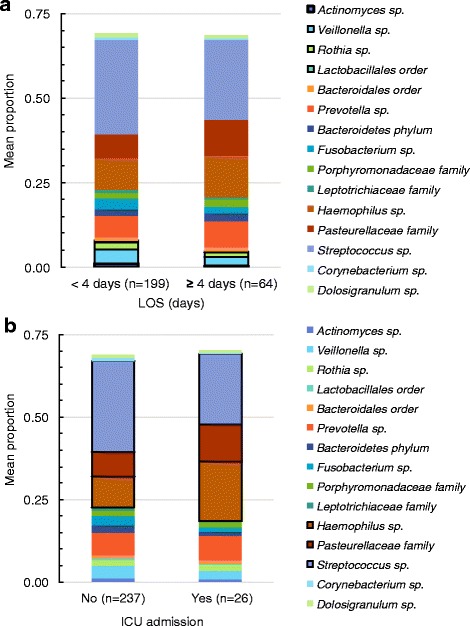
Mean proportions of taxa grouped by outcomes for subjects 6 months to <5 years of age length of hospital stay (LOS) (**a**) and intensive care unit (ICU) admission (**b**). Taxa of interest identified by principal component analysis (PCA) and associated with community-acquired pneumonia (CAP) severity are outlined in black

We identified four PCA factors in children 6 months to <5 years and two factors in children 5 to <18 years. Taxa within each factor and factor loadings are shown in Table 4. Figure 2 shows the mean proportion of individual taxa that were included in the factor analysis for subjects 6 months to <5 years grouped by LOS <4 days or ≥4 days (Fig. 2a) and ICU admission (Fig. 2b). Factor 1, which contained high relative abundance of *Actinomyces*, *Veillonella*, *Rothia* and Lactobacillales, was associated with decreased odds of LOS of ≥4 days [aOR 0.69; 95 % CI 0.48–0.99]. Factor 3, which contained high relative abundance of *Haemophilus* and Pasteurellacea and low relative abundances of *Streptococcus,* was associated with higher odds of ICU admission [aOR 1.52; 95 % CI 1.02–2.26].

**Table 4:** Rotated factor patterns from principal component analysis (PCA) using proportions of taxa in sputum samples from children 6 months to <5 years (*n* = 263) or 5 to <18 years (*n* = 120)

Taxa	Factor 1	Factor 2	Factor 3	Factor 4
Age 6 months to <5 years				
*Actinomyces* sp.	**0.84**	0.01	−0.11	−0.09
*Veillonella* sp.	**0.82**	0.21	−0.11	−0.12
*Rothia* sp.	**0.76**	−0.17	−0.05	0.03
*Lactobacillales* order	**0.64**	0.03	−0.10	−0.03
*Bacteroidales* order	0.08	**0.71**	−0.10	−0.05
*Prevotella* sp.	0.30	**0.63**	−0.05	−0.09
*Bacteroidetes* phylum	−0.16	**0.53**	−0.21	−0.12
*Fusobacterium* sp.	−0.08	**0.49**	0.01	−0.03
*Porphyromonadaceae* family	−0.02	**0.47**	−0.03	0.16
*Leptotrichiaceae* family	0.08	**0.41**	0.10	−0.16
*Haemophilus* sp.	−0.19	−0.18	**0.68**	−0.11
*Pasteurellaceae* family	−0.13	−0.19	**0.62**	−0.14
*Streptococcus* sp.	0.05	−0.33	**−0.79**	−0.25
*Corynebacterium* sp.	−0.03	−0.06	−0.03	**0.82**
*Dolosigranulum* sp.	−0.12	−0.12	−0.04	**0.81**
Age 5 to <18 years				
*Pasteurellaceae* family	**0.71**	−0.09		
*Haemophilus* sp.	**0.62**	0.00		
*Neisseria* sp.	**0.43**	−0.14		
*Rothia* sp.	**−0.47**	−0.30		
*Veillonella* sp.	**−0.57**	0.35		
*Porphyromonadaceae* family	−0.01	**0.69**		
*Bacteroidales* order	−0.15	**0.55**		
*Lactobacillales* order	−0.36	**0.48**		
*Prevotella* sp.	−0.34	**0.47**		
*Streptococcus* sp.	−0.36	**−0.62**		

Taxa of interest for each factor are in bold

**Fig. 3:**
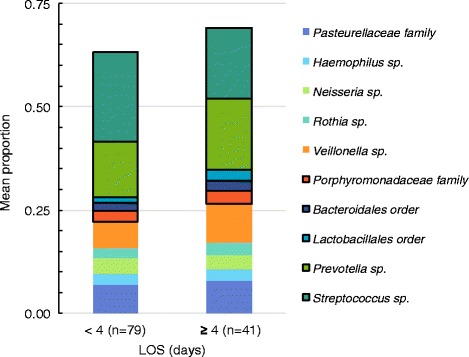
Mean proportion of taxa grouped by length of hospital stay (LOS) for subjects 5 to < 18 years. Taxa of interest identified by principal component analysis (PCA) and associated with LOS ≥4 days are outlined in black

Figure 3 shows the mean proportion of taxa included in the factor analysis of children ≥5 years grouped by LOS <4 days or ≥4 days. *Prevotella* and *Streptococcus* were two of the most abundant taxa in children ≥5 years. Factor 2 contained high relative abundance of Porphyromonadaceae, Bacteriodales, Lactobacillales, and *Prevotella* and low relative abundance of *Streptococcus*. In children ≥5 years, Factor 2 was associated with increased odds of a LOS ≥4 days [aOR 1.56; 95 % CI 1.03–2.36]. There were no linear combinations of taxa (PCA factors) that were significantly associated with ICU admission (data not shown).

### Adjusted associations between NP/OP microbiota profiles and CAP outcomes

Associations between microbiota profiles in NP/OP samples and CAP outcomes were evaluated in adjusted models similar to those described above for sputum samples.

#### Individual taxa in NP/OP samples

There were no differentially abundant taxa in NP/OP samples that were associated with LOS of ≥4 days or ICU admission in adjusted models (data not shown). Moreover, none of the individual taxa identified in sputum samples as associated with CAP severity were associated with LOS of ≥4 days or ICU admission in adjusted models when detected in NP/OP samples.

#### PCA factors in NP/OP samples

We examined microbiota profiles using PCA to identify relationships among correlated taxa in NP/OP samples. We identified three factors in children 6 months to <5 years and two factors in children 5 to <18 years. The correlated taxa in NP/OP PCA factors (Additional file 1: Table S4) differed from those identified in sputum samples (Table 4). There were no NP/OP PCA factors associated with CAP outcomes in adjusted models (data not shown).

## Discussion

Historically, researchers have viewed CAP as resulting from invasion of a single pathogen into the lungs. Recent data including the present analysis suggest that CAP should be considered as a polymicrobial disease. We described the association between bacterial taxa within the respiratory tract microbiota and severity of CAP. The relative abundance of specific taxa differed in induced sputum and NP/OP samples from children. None of the NP/OP measures of microbiota profiles were associated with CAP severity. In contrast, we identified individual taxa and three different community profiles (factors) in sputum samples that were associated with CAP severity. These data suggest that 1) induced sputum samples provide useful information for understanding the microbial environment of the respiratory tract in children hospitalized with CAP, and 2) high relative abundance and certain combinations of bacteria in the respiratory tract (e.g., Gram-negative taxa such as Porphyromonadaceae and *Prevotella*) may be linked to the clinical course of CAP in hospitalized children.

Commensals may contribute to the clinical course of CAP by altering host immune responses or the virulence of potential pathogens in a synergistic or additive manner [12–14]. Commensals may also produce β-lactamases that interfere with antibiotic treatment of respiratory tract infections due to physical proximity with pathogens [38–40]. In healthy individuals, distinct microbiota live in different body sites [41]. NP and OP samples were combined prior to 16S rRNA gene sequence analysis and we could not tell whether the taxa originated from an NP or OP site. Moreover, we cannot definitively determine whether our sputum samples contained predominantly lower respiratory tract bacteria or upper respiratory tract taxa acquired during sampling. However, microbiota studies indicate that: 1) the upper and lower respiratory tract form an interconnected and complex ecosystem; 2) there is erosion of the zones of demarcation that differentiate microbiota niches in diseased compared to healthy states; and 3) non-native resident taxa frequently and transiently invade interconnected body sites within the respiratory tract [5, 23, 42, 43]. Even if our induced sputum samples contained taxa from the upper respiratory tract, these taxa may still contribute to the microenvironment in the lung by production of metabolites and/or by stimulation of systemic host responses [23].

In children 6 months to <5 years, high relative abundance of *Actinomyces*, *Veillonella*, and *Rothia* were associated with a decreased odds of LOS ≥4 days each individually and as a group. *Actinomyces* and *Rothia* are both Gram-positive members of the family Actinomycetaceae and *Veillonella* is a Gram-negative anaerobe. These taxa are generally considered part of the normal flora but have been identified in individuals with CAP [7, 44–46]. High relative abundance of Gram-negative aerobes *Haemophilus* and Pasteurellacea and low relative abundance of *Streptococcus* were associated with ICU admission. Non-typeable *H. influenzae* has frequently been isolated from children with recurrent CAP [47]. However, *H. influenzae* was only designated as the etiology of CAP in one child in our study using standard diagnostic methods.

In children 5 to <18 years, high relative abundance of Gram-positive anaerobic *Gemella* species was associated with decreased odds of LOS ≥4 days. These species have been associated with pneumonia [48], but a negative association with severe CAP has not been appreciated previously. PCA analysis showed that high relative abundance of Porphyromonadaceae, Bacteriodales, Lactobacillales, and *Prevotella* and low relative abundance of *Streptococcus* were associated with increased odds of LOS ≥4 days in children ≥5 years. Gram-positive *Lactobacillales* (i.e., lactic acid bacteria) are generally associated with respiratory health [9]. However, Gram-negative anaerobes including *Porphyromonas* species, Bacteroides, and pigmented *Prevotella* have been detected in cases of aspiration pneumonia and empyema in children [49]. When these taxa are identified in children with pneumonia, they are often found in mixed infections [50]. *Prevotella intermedia,* an oral bacterium, was recently shown to have synergistic effects on *S. pneumoniae* virulence in murine models of bacteremic pneumonia [51]. Transtrachial aspiration followed by culture has been used to identify anaerobes in 22–33 % of adults with CAP [52]. Thus, Gram-negative anaerobes such as *Prevotella* may play an important and underappreciated role in the clinical course of CAP in older children.

A natural question is whether 16S rRNA microbial profiling adds important information relative to culture-based protocols. Log transformed relative abundances of *Streptococcus*, *Heamophilus*, *Moraxella*, and *Staphylococcus* were significantly associated with identification of *S. pneumoniae*, *H. influenzae*, *M. cattharalis*, and *S. aureus* by culture (*P* = <0.0001 for each species identified by culture and the corresponding genus level taxon as determined by *t*-test). Culture of *S. aureus* was associated with increased odds of a LOS ≥4 days [aOR 2.48; 95 % CI 1.25–4.90] in children 6 months to <5 years (Additional file 1: Table S3). Culture of the other bacterial pathogens from sputum was not associated with CAP severity (Additional file 1: Table S3). Thus, our data suggest that important insights can be gained by culture-independent analysis of the microbial community in sputum samples from children with CAP.

Our study has limitations and any potential causal associations between the presence of the identified taxa and CAP severity must be interpreted with caution. Induced sputum samples were collected once and we could not evaluate temporal changes in the microbiota. Contamination in 16S rRNA microbial profiling and metagenomic studies has been linked previously to water- and soil-associated bacteria [53]. We did not sequence reagent controls and there was potential for contamination despite our use of molecular biology grade water and commercial kits [53]. While we cannot definitively rule out contamination, we have several lines of evidence to suggest that our data reflect the community composition of our clinical samples: 1) samples were processed in random order, PCR cycles were kept to a minimum, and PCR reactions were done in duplicate as recommended by Salter et al. [53]; 2) we limited our analyses to taxa present in greater than 0.8 % of the microbial community and did not include rare taxa in our analyses; and 3) our dominant taxa are consistent with those generally identified in studies of the human respiratory tract [11, 26, 27]. We did not calculate alpha and beta diversity measures because of concerns regarding the potential for inflation of diversity estimates due to contamination, PCR amplification, and sequencing errors on the Illumina platform [54, 55]. Our study population had high a proportion of children with asthma/reactive airway disease and other chronic conditions and our results may not be generalizable to all hospitalized children with CAP.

An improved understanding of the role of the respiratory microbiota in CAP may yield novel approaches towards prevention or treatment. For example, strategies for selectively introducing commensals (e.g., microbiota transplants) to promote optimal levels of biodiversity in the airways may emerge as effective methods for treating disease. However, more data are needed regarding the role of the respiratory microbiota in the clinical course of CAP before the translational goals of manipulating the respiratory flora for optimal health and resolution of disease can be realized. Additional studies are needed to confirm our findings and to provide mechanistic insights into relationships between the respiratory tract microbiota and CAP. Future data from laboratory and prospective epidemiologic studies will facilitate the design of effective prevention and treatment strategies for CAP that maintain the health-associated homeostasis between children and their commensal flora.

## Conclusions

Our data suggest that sputum samples are useful for understanding the microbial environment in the respiratory tract during community-acquired pneumonia. Microbiota profiles associated with severity of pneumonia differed in children 6 months to <5 years compared to children 5 to <18 years of age. Specific combinations of bacteria in the respiratory tract may influence the clinical course of pneumonia; high abundance of some bacteria, predominantly Gram-negative species, was associated with increased severity of pneumonia.

## Abbreviations

CAP, community-acquired pneumonia; CDC, centers for disease control; ICU, intensive care unit; LDA, linear discriminant analysis; LefSe, linear discriminant analysis effect size; LOS, length of stay; NP, nasopharyngeal; OP, oropharyngeal; PCA, principal component analysis; RSV, respiratory syncytial virus

## Additional files

Additional file 1: Table S1. Value for the top quartile (75^th^ percentile) for frequency distributions of taxa stratified by subject age for sputum and nasopharyngeal/oropharyngeal (NP/OP) samples. **Table S2.** Diagnostic tests for detection of respiratory viruses and bacteria [n (%)] in children (*n* = 383) 6 months to <18 years of age hospitalized with community-acquired pneumonia by age group. **Table S3.** Adjusted associations between sputum culture results from children 6 months to <5 years (*n* = 263) or 5 to <18 years (*n* = 120), hospitalized with community-acquired pneumonia and length of stay (LOS) or intensive care unit (ICU) admission.** Table S4.** Rotated factor patterns from principal components analysis (PCA) using proportions of taxa in nasopharyngeal/oropharyngeal (NP/OP) samples from children 6 months to <5 years (*n* = 263) or 5 to <18 years (*n* = 120). (PDF 86 kb) Additional file 2: Figure S1. Proportion of children with a given dominant bacterial taxon. (PDF 273 kb)
