# Supplementary material for: Association of sputum microbiota profiles with severity of community-acquired pneumonia in children
Source: BMC Infect Dis. 2016 Jul 8;16:317. doi: 10.1186/s12879-016-1670-4 (PMC4939047; doi:10.1186/s12879-016-1670-4)
Supplement: Additional file 1: Table S1. — Value for the top quartile (75th percentile) for frequency distributions of taxa stratified by subject age for sputum and nasopharyngeal/oropharyngeal (NP/OP) samples. Table S2. Diagnostic tests for detection of respiratory viruses and bacteria [n (%)] in children (n = 383) 6 months to <18 years of age hospitalized with community-acquired pneumonia by age group. Table S3. Adjusted associations between sputum culture results from children 6 months to <5 years (n = 263) or 5 to <18 years (n = 120), hospitalized with community-acquired pneumonia and length of stay (LOS) or intensive care unit (ICU) admission. Table S4. Rotated factor patterns from principal components analysis (PCA) using proportions of taxa in nasopharyngeal/oropharyngeal (NP/OP) samples from children 6 months to <5 years (n = 263) or 5 to <18 years (n = 120). (PDF 86 kb) [file 12879_2016_1670_MOESM1_ESM.pdf]

Supplemental Table 1. Value for the top quartile (75th percentile) for frequency distributions of taxa stratified by subject age for sputum and nasopharyngeal/oropharyngeal (NP/OP) samples[a].

	**Age < 5 years**		**Age ≥ 5 years**	
**OTU**	**Sputum**	**NP/OP**	**Sputum**	**NP/OP**
Actinomyces sp.	1.49	3.30	3.02	4.17
Atopobium sp.	0.17	0.93	0.58	1.70
Bacteroidales order	1.30	1.04	2.82	2.38
Bacteroidetes phylum	2.45	1.00	1.61	0.65
Corynebacterium sp.	0.49	1.18	0.59	1.34
Dolosigranulum sp.	0.55	1.14	0.00	0.96
Fusobacterium sp.	3.07	1.25	6.44	2.28
Gemella sp.	1.32	1.88	2.02	1.61
Haemophilus sp.	10.63	1.68	1.32	0.17
Lactobacillales order	0.74	2.33	1.91	2.03
Leptotrichia sp.	0.90	0.84	2.99	1.27
Leptotrichiaceae family	0.86	0.66	0.96	0.45
Moraxella sp.	27.89	5.77	0.19	1.07
Mycoplasma sp.	0.00	0.00	0.10	0.02
Neisseria sp.	3.06	2.03	4.50	1.28
Pasteurellaceae family	10.01	3.52	8.79	1.91
Porphyromonadaceae family	1.98	1.35	3.70	1.97
Prevotella sp.	9.57	13.50	21.04	21.69
Rothia sp.	1.78	8.75	3.32	4.17
Streptococcus sp.	38.65	46.89	27.32	30.69
Veillonella sp.	5.07	10.92	10.55	12.58
Other	12.10	12.04	18.92	23.19

[a]Table values represent a percent, e.g., the relative abundance of *Veillonella* sp. was at least 5.07% of the sample in approximately 25% of the sputum samples for children 6 months to <5 years of age.

Supplemental Table 2. Diagnostic tests for detection of respiratory viruses and bacteria [n (%)] in children (n=383) 6 months to <18 years of age hospitalized with community-acquired pneumonia by age group

**Characteristic**	**N (%)**	**Age group**		**P value**
		**6 months to <5 years (%)**	**5 to <18 years (%)**	
**Total**	383	263(68.7)	120(31.3)	
Respiratory viruses				
Rhinovirus	148 (38.6)	33.8	49.2	0.004
RSV	123 (32.1)	41.8	10.8	<0.0001
Adenovirus	61 (15.9)	21.3	4.2	<0.0001
Human metapneumovirus	26 (7.0)	9.1	1.7	0.007
Coronavirus	24 (6.3)	7.6	3.3	0.11
Parainfluenza types 1-3	27 (7.0)	8.4	4.2	0.14
Influenza A and B	17 (4.4)	3.8	5.8	0.37
Bacteria diagnostic tests				
M. pneumoniae	14 (3.7)	1.5	8.3	0.001
S. pneumoniae	9 (2.4)	3.4	0.0	0.06
H. influenzae	1 (0.3)	0.4	0.0	--
Bacteria sputum culture				
S. pneumoniae	64 (16.7)	22.4	4.2	<0.0001
H. influenzae	47 (12.3)	16.0	4.2	0.001
M. cattharalis	65 (17.0)	23.9	1.7	<0.0001
S. aureus	68 (17.8)	17.1	19.2	0.63

Supplemental Table 3. Adjusted associations between sputum culture results from children 6 months to <5 years (n=263) or 5 to <18 years (n=120), hospitalized with community-acquired pneumonia and length of stay (LOS) or intensive care unit (ICU) admission.

	**LOS ≥ 4 days**	**ICU admission**
**Characteristic**	**OR (95% CI)**	**OR (95% CI)**
**6 months to <5 years**		
S. pneumoniae	0.97 (0.49, 1.93)	0.56 (0.18, 1.74)
H. influenzae	1.97 (0.97, 4.02)	1.02 (0.11, 0.85)
M. cattharalis	1.09 (0.57, 2.11)	0.57 (0.15, 0.46)
S. aureus	2.48 (1.25, 4.90)	1.52 (0.56, 4.15)
**5 to <18 years**		
S. aureus	1.28 (0.46, 3.56)	1.81 (0.63, 5.20)

Supplemental Table 4. Rotated factor patterns from principal components analysis (PCA) using proportions of taxa in nasopharyngeal/oropharyngeal (NP/OP) samples from children 6 months to <5 years (n=263) or 5 to <18 years (n=120). Taxa of interest for each factor are highlighted.

Taxa	Factor 1	Factor 2	Factor 3
**Age 6 months to <5 years**			
Prevotella sp.	0.75	0.16	-0.14
Veillonella sp.	0.72	-0.20	-0.30
Actinomyces sp.	0.70	-0.18	-0.17
Leptotrichiaceae family	0.69	0.21	0.08
Atopobium sp.	0.59	-0.27	-0.16
Bacteroidales order	0.32	0.67	-0.09
Bacteroidetes phylum	-0.15	0.63	-0.15
Fusobacterium sp.	0.34	0.58	0.20
Porphyromonadaceae family	-0.13	0.47	-0.12
Neisseria sp.	-0.10	0.45	-0.06
Corynebacterium sp.	-0.09	-0.08	0.75
Dolosigranulum sp.	-0.09	-0.09	0.83
Moraxella sp.	-0.24	-0.12	0.53
**Age 5 to <18 years**			
Prevotella sp.	0.74	-0.29	
Veillonella sp.	0.71	-0.16	
Atopobium sp.	0.65	-0.31	
Actinomyces sp.	0.52	-0.07	
Bacteroidales order	0.47	-0.18	
Corynebacterium sp.	-0.43	-0.24	
Moraxella sp.	-0.53	-0.29	
Dolosigranulum sp.	-0.61	-0.35	
Pasteurellaceae family	-0.13	0.76	
Neisseria sp.	-0.07	0.66	
Gemella sp.	0.03	0.54	
Haemophilus sp.	-0.07	0.53	
