# Supplementary figures and images for: Association of sputum microbiota profiles with severity of community-acquired pneumonia in children
Source: BMC Infect Dis. 2016 Jul 8;16:317. doi: 10.1186/s12879-016-1670-4 (PMC4939047; doi:10.1186/s12879-016-1670-4)

**Supplemental Figure 1.** Proportion of children with a given dominant bacterial taxon

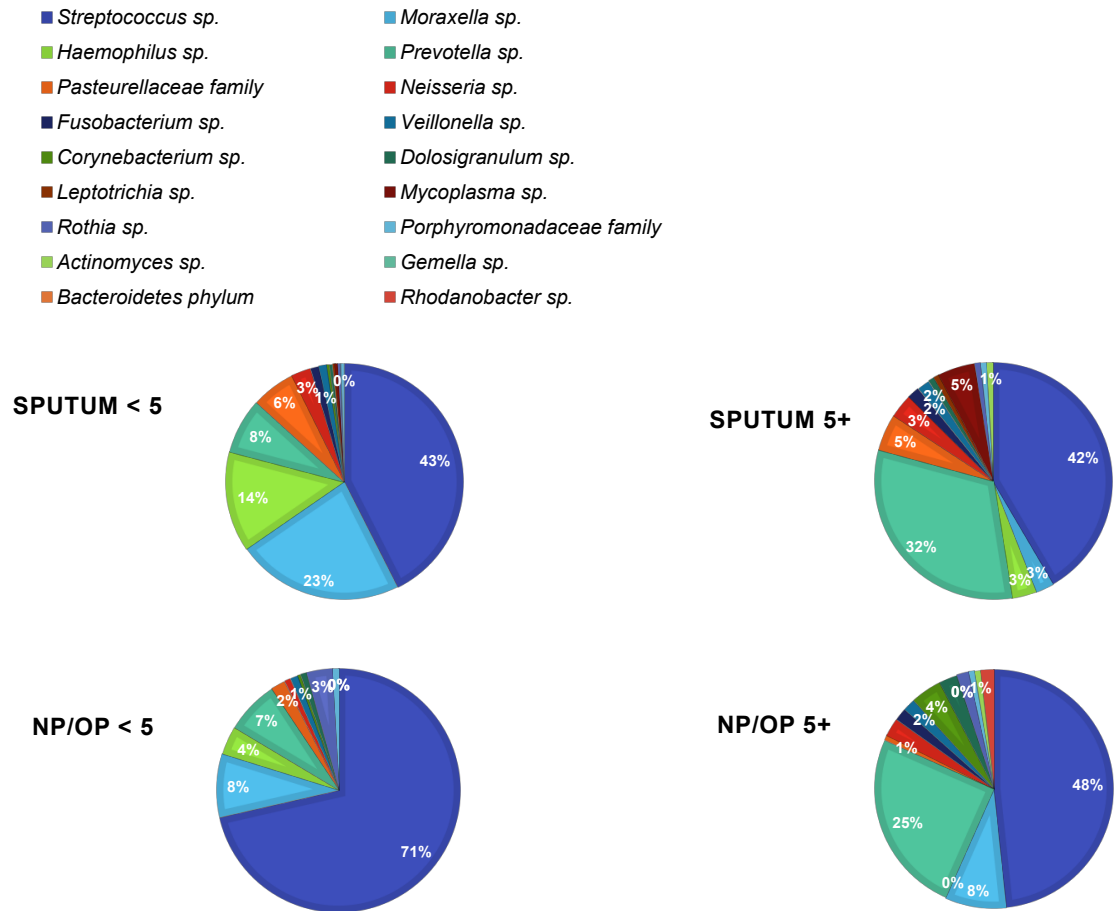

Supplement: Additional file 2: Figure S1. — Proportion of children with a given dominant bacterial taxon. (PDF 273 kb) [file 12879_2016_1670_MOESM2_ESM.pdf]
